# Alcohol intake patterns, stress exposures and oral health in treatment seeking individuals with alcohol use disorder

**DOI:** 10.3389/froh.2026.1736280

**Published:** 2026-05-08

**Authors:** Chelsea B. Crayton, Jennifer J. Barb, Li Yang, Christian Otado Mbulu, Melanie L. Schwandt, Sharon Sawyer, Kimberly Herman, Vijay A. Ramchandani, Nancy Diazgranados, Gwenyth R. Wallen, Katherine A. Maki

**Affiliations:** 1 Translational Biobehavioral and Health Promotion Branch, Clinical Center, National Institutes of Health, Bethesda, MD, United States; 2 National Institute on Alcohol Abuse and Alcoholism, National Institutes of Health, Bethesda, MD, United States

**Keywords:** adverse childhood experiences, alcohol use, alcohol use disorder, dental care, health outcomes, oral health, periodontal disease, stress/psychological

## Abstract

**Background:**

Alcohol use and stress are linked to negative oral health outcomes, but data are limited in individuals with alcohol use disorder (AUD). This study examines alcohol consumption, perceived stress, adverse childhood experiences (ACEs), and oral health in individuals with AUD.

**Methods:**

In this retrospective analysis, oral health, alcohol use, perceived stress, and childhood trauma data were abstracted from existing records for treatment-seeking inpatients with AUD who were referred for dental examination (*N* = 99). Multivariable models adjusted for available demographic and clinical covariates. Data on oral hygiene practices, diet, and access to dental care were not available.

**Results:**

Seventy-three percent of the individuals with AUD had moderate-severe periodontal disease (PD), and 30% reported oral pain. Higher PD severity was associated with increased years of heavy drinking (*p* < .001). Perceived stress was significantly associated with PD severity (*p* < .001), while ACE exposures were linked to oral pain (*p* = .031). After controlling relevant variables, decreased perceived stress and increased ACEs remained significantly associated with increased PD severity, while heavy drinking years did not. In the fully adjusted model, smoking was significantly associated with an increased likelihood of oral pain.

**Conclusion:**

In this referred inpatient sample, psychosocial variables showed associations with oral health severity after adjustment for available covariates. This suggests individuals with AUD are at high risk for poor oral health, but factors beyond alcohol consumption may influence this relationship. Nevertheless, the retrospective design, limited sample size, and unavailable oral health behavior data warrant cautious interpretation. Integrating assessment of past and current stress exposures, along with increased referral for dental evaluation, could improve whole person AUD treatment and health outcomes in individuals with AUD.

## 1 Introduction

Alcohol misuse is a significant global public health issue, contributing to 3 million deaths annually (1). In the United States, over 25% of adults have reported binge drinking, and one-third have met the DSM-5 criteria for Alcohol Use Disorder (AUD) at some point in their lives (2–5). AUD is characterized by an individual's inability to stop or control alcohol use despite facing social, economic, and health consequences (3, 6). Individuals with AUD often develop a physiological dependency on alcohol, experiencing increased tolerance, cravings, and withdrawal cycles (4, 7). Long-term or heavy alcohol consumption is linked to higher markers of systemic inflammation and a greater risk of chronic disease (2–4). In 2019, the economic cost of AUD in the United States was estimated at $249 billion, with healthcare costs making up $27 billion of this total (3, 6). As the prevalence of AUD rises, it is crucial to understand its impact on various body systems and the associated public health risks, given its significant contribution to morbidity and healthcare costs.

Oral health includes the condition of an individual's mouth, teeth, and orofacial structures, which are essential for daily activities such as eating, speaking, and breathing (8). Problems like tooth loss, periodontal disease (PD), and general oral deterioration can hinder these activities (9–11), and negatively impact self-esteem (12, 13). Currently, approximately 42% of United States adults aged 30 years or older have some form of PD (14), and 26% have untreated tooth decay (15). Barriers to accessing preventative oral health care, such as low household income or the absence of dental coverage in many standard private and public insurance plans potentially exacerbate the risk for dental or PD in the context of health behaviors like cigarette smoking or heavy alcohol use (16). Chronic alcohol consumption is connected to profound changes in the oral environment including decreased oral pH, decreased saliva production, and disrupted tooth enamel (17–19). Chronic alcohol use leads to an increase in the development and progression of dental caries and tooth loss, likely accelerated due to the high sugar content in alcoholic beverages (17, 20).

Emerging evidence suggests that stress exposures are associated with increased oral complaints and PD (21, 22), further compounding the oral health risks associated with AUD, including alcohol-related changes in the oral environment and psychosocial responses to alcohol intoxication and withdrawal. Described as the thoughts, feelings, and perception of an individual regarding their stress load over a set period of time, perceived stress is commonly used to assess the relationship between stress and health outcomes (23). Stress manifests in oral health pathology (e.g., increased caries and PD severity) in children and adults, suggesting that stressors may act as measurable health outcomes that increase an individual's risk for disease (22, 24, 25).

**Figure 1:**
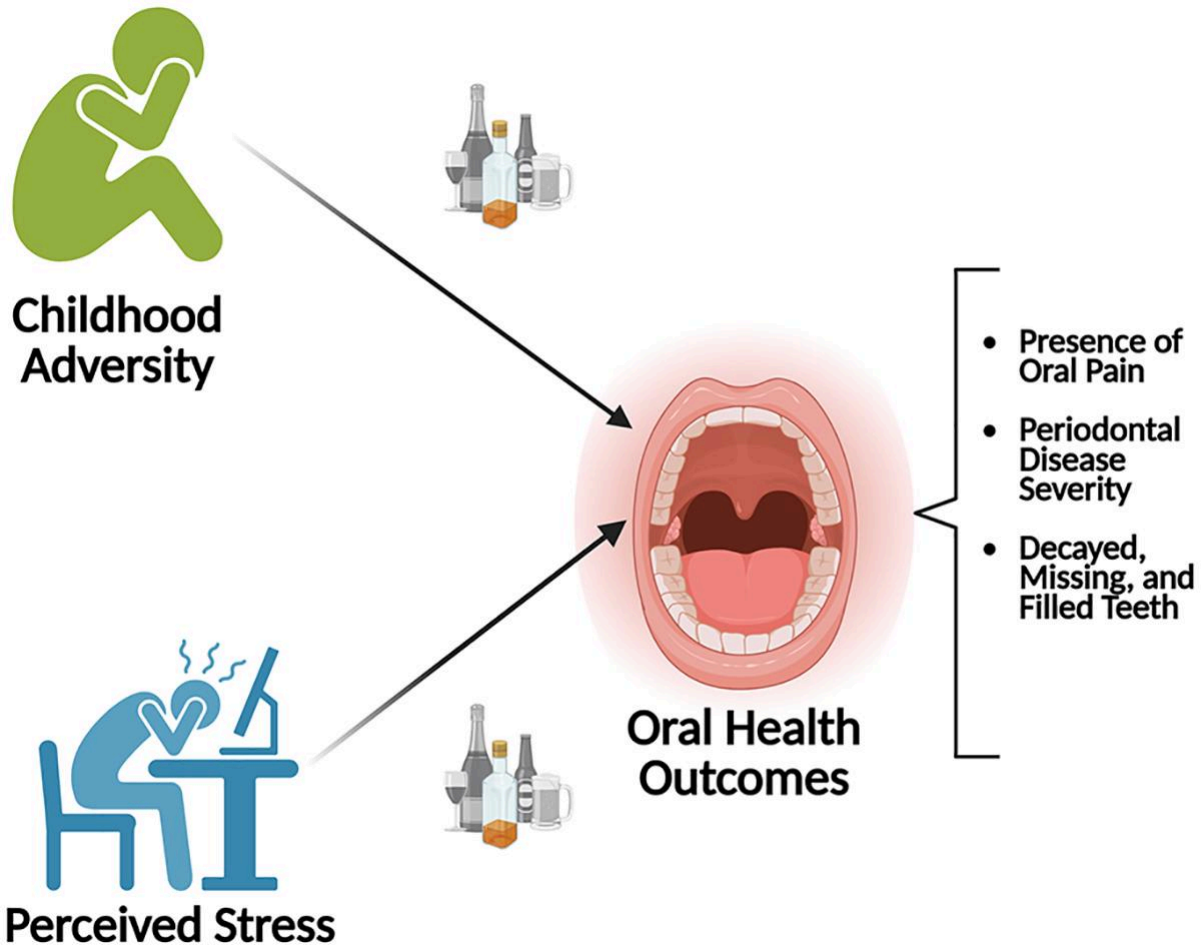
Proposed study model. Schematic of the conceptual model guiding the study design and analysis framework. Perceived stress severity and increased exposure to ACEs are suspected to influence the objective and subjective oral health characteristics in patients with AUD. Created in BioRender. Maki, K. (2025) https://BioRender.com/lzp36qr.

Elevated perceived stress levels are also associated with increased episodes of binge drinking, number of daily drinks consumed, and frequency of drinking behaviors in young adults, which may amplify the risk for oral disease due to interactions between heavy or chronic alcohol use with the mouth (26, 27). Studies examining the relationship between perceived stress and substance use severity, age or relapse risk among individuals with substance use disorders have reported mixed findings. Some studies suggest that perceived stress may predispose individuals to relapse following inpatient treatment (28, 29), and may be associated with later or repeated alcohol use (30), whereas others have found no significant relationship between alcohol use and perceived stress symptoms (30). Although the current literature has reported associations between perceived stress and alcohol use, limited research has evaluated adverse childhood experiences (ACEs) as an additional measure of stress exposure that may capture the longer-term effects of stress on current perceived stress and oral health (Figure 1).

ACEs are potentially traumatic events that occur during childhood and early adolescence (before the age of 18), and common ACEs are witnessing violence at home or within a community, experiencing neglect or abuse, and losing of a family member (31). Six in 10 United States adults have experienced at least one ACE, with nearly 20% experiencing four or more (31). Experiencing an ACE has long-term impacts on an individual's life that include reporting higher levels of perceived stress in early adulthood (32–34), increasing the risk of early initiation of alcohol use and developing AUD (35, 36). In both treatment-seeking and non-treatment-seeking populations, experiencing ACEs predict higher rates of alcohol dependency (37–40). Similarly, ACEs are associated with long-term negative outcomes in oral health and oral wellbeing (41, 42). Previous studies in the United States, have found that experiencing an ACE is associated with infrequent dental cleanings (41, 43), lower likelihood of having annual dental check-ups (43), and an increased risk of having six or more permanent teeth extracted (41, 43).

Although there is research linking alcohol use to oral decay and poor dentition, a comprehensive evaluation of oral health status and stress in treatment seeking patients with AUD has yet to be explored. Therefore, this retrospective analysis aims to use data collected as part of a natural history study in treatment-seeking individuals with AUD to explore relationships between ACEs, perceived stress levels prior to inpatient admission, and oral health outcomes. We hypothesized that: (1) Alcohol use chronicity would be associated with increased PD severity and incidence of oral pain; (2) Participants with higher reported Perceived Stress Scale (PSS) scores prior to inpatient treatment for AUD would have greater PD severity and incidence of oral pain; and (3) Participants who reported increased ACE exposures would have higher rates of PD severity or oral pain complaints.

## 2 Methods

### 2.1 Study design and data collection

This is a retrospective analysis of treatment-seeking individuals with AUD who underwent 4 weeks of inpatient treatment at the National Institutes of Health (NIH) Clinical Center, and the data on which this analysis is based was collected using the NIH medical records systems. Through a joint effort between investigators at the NIH Clinical Center, the National Institute on Alcohol Abuse and Alcoholism (NIAAA), and the National Institute of Dental and Craniofacial Research (NIDCR), data was collated from the Clinical Research Information System and Biomedical Translational Research Information System (internal electronic health record systems) and the Eaglesoft Dental Practice Management Software (Patterson Dental Supply, Inc., St. Paul, MN) to compile relevant clinical dental data.

### 2.2 Sample of participants

**Figure 2:**
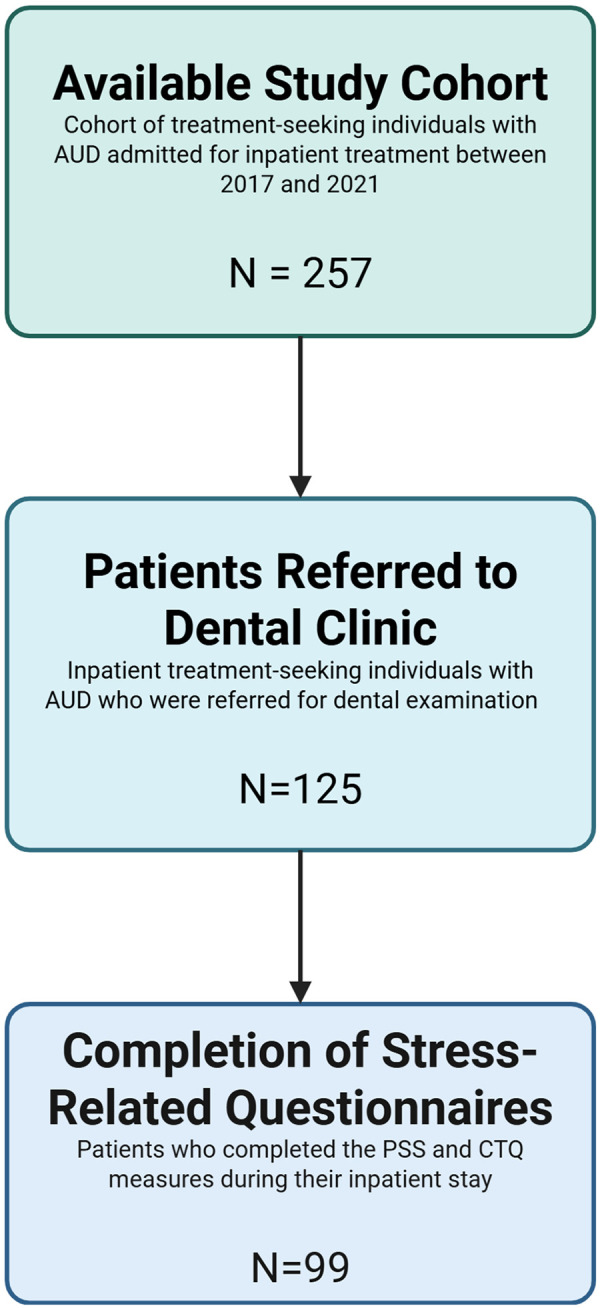
Study sample filtering flowchart. Legend: The original sample included for retrospective analysis of treatment-seeking individuals with alcohol use disorder admitted to the NIH Clinical Center was *N* = 257. Filtering individuals without dental referrals and no periodontal disease severity examination, removed 132, leaving a samples size of *N* = 125. Finally, participants with missing perceived stress scale (PSS) and/or childhood trauma questionnaire (CTQ) data were excluded resulting in a final sample size for analysis of *N* = 99. Created in BioRender. Maki, K. (2025) https://BioRender.com/lzp36qr.

The available sample cohort included inpatient treatment-seeking individuals diagnosed with AUD using DSM-5 criteria or alcohol dependence using DSM-IV criteria following administration of The Structured Clinical Interview for DSM-IV (44, 45) or DSM-5 (46, 47). Patients who were admitted to the NIH Clinical Center between January 2017 and December 2021 were screened for inclusion in this retrospective analysis (*n* = 257). All study participants provided written informed consent under a NIAAA Natural History Protocol approved by the NIH institutional review board in accordance with the Declaration of Helsinki (ClinicalTrials.gov Registry Number NCT02231840; Registration date 9/3/2014). Subjects who were included in this analysis received a dental and periodontal examination during the inpatient treatment period and must have completed both stress evaluation instruments [PSS and Childhood Trauma Questionnaire (CTQ) assessments]. Of the 257 inpatients, 49% were referred for dental examination (*n* = 125), and after screening out patients without PSS or CTQ, a total of 99 patients were studied (Figure 2). Patients with multiple admissions to the Clinical Center were included, however, only data from their most recent visit were used in the final data set.

### 2.3 Demographics

Patient demographic information included characterizing age, sex, race and ethnicity, years of education and income level. Endorsement of current smoking status was recoded to categorize patients into current smokers or non-smokers. Detailed demographic information of the study sample population is depicted in Table 1. Demographic, alcohol, and stress-associated variables in subjects referred for dental examination (study sample population) vs. not referred for dental examination can be found in Supplementary Table S1.

**Table 1:** Demographic and clinical characteristics of participants stratified by periodontal disease severity.

Variables		Total Sample	PD Assessment Groups			*p*
			Gingivitis/Mild PD	Moderate PD	Severe PD	
		(*n* = 99)	(*n* = 26)	(*n* = 44)	(*n* = 29)	
Sociodemographic Characteristics of Sample Population						
Age (Mean ± SD)		45.89 ± 11.3	37.92 ± 9.1	44.86 ± 9.9	54.59 ± 9.1	**<.001** ^a^
Sex, N (%)	Male	69 (69.7)	15 (57.7)	29 (65.9)	25 (86.2)	.055^b^
	Female	30 (30.3)	11 (42.3)	15 (34.1)	4 (13.8)	
Race & Ethnicity, N (%)	Non-Hispanic White	51 (51.5)	17 (65.4)	22 (50.0)	12 (41.4)	.073^b^
	Non-Hispanic Black	31 (31.3)	5 (19.2)	11 (25.0)	15 (51.7)	
	Other	6 (6.1)	2 (7.7)	4 (9.1)		
	Hispanic/Latino	11 (11.1)	2 (7.7)	7 (15.9)	2 (6.9)	
Marital Status, N (%)	Single	53 (53.5)	2 (7.7)	23 (52.3)	14 (48.3)	.914^b^
	Married	10 (10.1)	16 (61.5)	5 (11.4)	3 (10.3)	
	Divorced/Separated/ Widowed	29 (29.2)	8 (30.8)	11 (25.0)	10 (34.5)	
	Not Specified	7 (7.1)		5 (11.4)	2 (6.9)	
Education Level, N (%)	Elementary School	3 (3.0)		1 (2.3)	2 (6.9)	.300^b^
	High School	39 (39.4)	7 (26.9)	17 (38.6)	15 (51.7)	
	College	49 (49.5)	17 (65.4)	22 (50.0)	10 (34.5)	
	Graduate School or Higher	8 (8.1)	2 (7.7)	4 (9.1)	2 (6.9)	
Household Income, N (%)	< $5,000	27 (27.3)	7 (26.9)	9 (20.5)	11 (37.9)	.071^b^
	$5,000 –$19,000	25 (25.3)	7 (26.9)	9 (20.5)	9 (31.0)	
	$20,000–$49,999	25 (25.3)	7 (26.9)	10 (22.7)	8 (27.6)	
	$50,000–>$100,000	22 (22.2)	5 (19.2)	16 (36.4)	1 (3.4)	
Smoking Status, N (%)	Smoker	64 (64.6)	13 (50.0)	29 (65.9)	22 (75.9)	.131^b^
	Non-smoker	35 (35.4)	13 (50.0)	15 (34.1)	7 (24.1)	
Stress Characteristics of Sample Population						
PSS Total Score (Mean ± SD)		22.78 ± 7.53	27.38 ± 6.89	21.41 ± 7.24	20.72 ± 6.97	**<.001** ^a^
CTQ Total Score (Mean ± SD)		42.84 ± 20.65	38.23 ± 15.77	46.68 ± 21.55	41.14 ± 22.61	.223^a^
Alcohol Use Profile of Sample Population						
*Average Drinks Per Day (Mean ± SD)*		17.34 ± 12.15	20.18 ± 15.24	16.00 ± 9.32	16.81 ± 12.85	.369^a^
*Heavy Drinking Years (Mean ± SD)*		17.52 ± 11.87	11.61 ± 8.53	14.55 ± 8.86	27.14 ± 12.87	**<.001** ^a^
Dental Disease and Oral Pain of Sample Population						
*Decayed, Missing, and Filled Teeth (Mean ± SD)*		10.21 ± 7.60	7.08 ± 8.03	10.13 ± 5.72	12.93 ± 8.59	**.017** ^a^
*Oral Pain Presence, N (%)*	Yes, Pain	30 (30.3)	6 (23.1)	14 (31.8)	10 (34.5)	.614^b^
	No Pain	43 (43.4)	13 (50.0)	18 (40.9)	12 (41.4)	
	Not Reported	26 (26.3)	7 (26.9)	12 (27.3)	7 (24.1)	

a Statistics Completed using ANOVA.

b Statistics Completed using Pearson Chi-square test.

PD, periodontal disease, SD, standard deviation.

Values in bold indicate statistically significant *p* values.

### 2.4 Alcohol use characteristics

The average amount of alcohol intake and chronicity of heavy alcohol use was assessed using information gathered from The Alcohol Timeline Follow Back (TLFB) and the Lifetime Drinking History (LDH) questionnaires. The TLFB is a standard assessment for measuring alcohol drinking patterns and quantification in treatment programs for individuals with AUD and collects drinking information using personal historical events that are recounted over the previous 90-day period (48, 49) from the date of assessment. Outcome measures include total number of drinks over 90-days prior to treatment, the number of drinking days, the number of heavy drinking days, and the average number of drinks per drinking day. The LDH is a structured interview designed to provide quantitative indices of an individual's alcohol consumption patterns from the onset of regular drinking (50). Average drinks per day in the 90 days prior to admission (via the TLFB) and number of heavy drinking years (via the LDH) were used as the alcohol intake variables in the final analysis.

### 2.5 Perceived stress

Perceived stress was assessed using the PSS. The PSS is a 10-item self-report psychological measure used to examine one's perception of stress over the last 30 days (51), and average perceived stress over the 30 days prior to admission for inpatient treatment was quantified. Scored on a 5-point Likert scale (0 = never, 1 = almost never, 2 = sometimes, 3 = fairly often, 4 = very often), the PSS can range from total scores of 0 to 40, which are operationalized as low severity (0–13), moderate severity (14–26), and high severity (27–40) of PSS (51).

### 2.6 Adverse childhood experiences

To evaluate ACEs exposure in this population, past traumatic experiences were assessed by the CTQ. The CTQ is used to examine the occurrence and frequency of ACEs prior to the age of 18 (52), and specifically focuses on five domains of traumatic experiences: emotional abuse, physical abuse, sexual abuse, emotional neglect, and physical neglect. Each domain is represented by five questions rated on a 5-point Likert scale (1 = never true, 2 = rarely true, 3 = sometimes true, 4 = often true, 5 = very often true) to produce sub-domain severity scores that can range from 5 to 25. Total scores for the CTQ range from 25 to 125 and is calculated by adding the sum score of five domains of traumatic experiences (53). CTQ scores greater than 35 are considered a clinically significant history of childhood trauma in previous reports (54).

### 2.7 Objective oral health measures

#### 2.7.1 Periodontal disease (PD) profiling

Classification of the extent of PD was created for each participant using findings reported by the NIDCR dental clinic. PD severity was abstracted retrospectively from routine dental records generated during clinical care at the NIDCR dental clinic in collaboration with the dental team. Categories were based on clinician-documented diagnoses recorded in dental progress notes and associated imaging reports available in the medical record. For analysis, participants were grouped as gingivitis/mild PD, moderate PD, or severe PD. Gingivitis and mild periodontitis were combined because the number of participants with gingivitis diagnoses alone was low (*n* = 8), which limited stable subgroup comparisons (See Supplementary Table S2 for sub-diagnosis distribution). These grouped categories were created to support statistical analyses and should not be interpreted as indicating clinical equivalence between gingivitis and mild periodontitis. Because PD severity categories were derived from routine clinical documentation rather than a prospectively standardized research examination, examiner calibration and inter-clinician reliability data were not available.

#### 2.7.2 Retrospective decayed, missing, and filled teeth estimate

The number of decayed, missing, and filled teeth (DMFT) were retrospectively captured using existing scores from the DMFT-28 scores when documented, dental clinic charting, and radiographic findings. For some participants, a DMFT value had already been recorded in the clinical dental record; for others, a DMFT estimate was generated through retrospective chart abstraction using written dental documentation and panoramic radiographic findings available in the medical record. Teeth classified as decayed included those documented as caries, root tips, periapical abscess, and/or distal decay in dental charting. Missing teeth included those recorded as missing or extracted. Filled/restored teeth included those documented as restorations, root canal treatment or as a part of a bridge. Because these data were derived or abstracted retrospectively from routine clinical documentation rather than obtained through a prospectively standardized research assessment, this variable is referred to throughout the manuscript as a DMFT estimate. This terminology is intended to reflect the chart-derived nature of the measure and to avoid overinterpretation of findings based on retrospectively abstracted dental documentation.

#### 2.7.3 Oral pain

Self-reported presence of any oral pain was obtained through intake notes from the dental clinic. Characteristics or type of the pain reported, and location of pain was not included in the notes provided to this team. If patient reported oral pain at any severity, they were coded as “yes” and if a patient did not report oral pain they were coded as “no”.

### 2.8 Data analysis

Descriptive statistics (mean and standard deviation for continuous data, frequencies, and percentages for categorical data) were used to describe the sample demographics, alcohol usage, PSS and CTQ scores, and oral health measures. Relationships between individual variables were examined with bivariate methods including Pearson correlations, Chi-square tests, independent *t*-tests, and analysis of variance (ANOVA) models. Multinomial logistic regression models were used to assess the impact of demographic, alcohol-related, and stress-associated variables with objective (DMFT estimate and PD severity) oral health measures, with the gingivitis/mild PD severity group as the reference category for PD severity outcomes. Binary logistic regression was used to examine factors associated with oral pain among participants with reported oral pain status. Covariates were selected *a priori* based on clinical relevance and availability in reviewed charts, and included age, sex, smoking status, and household income; the PD severity model additionally included heavy drinking years, PSS score, and CTQ score. Adjusted odds ratios (ORs) and 95% confidence intervals (CIs) are reported for all multivariable models. Data analyses were conducted using IBM SPSS statistics version 29 and JMP Statistical Discovery Software (version 16, SAS Headquarters, Cary, NC). Figures were generated using the JMP Statistical Discovery Software. A *p-*value less than .05 was considered statistically significant.

## 3 Results

### 3.1 Study population

Patients' sociodemographic characteristics, alcohol consumption, and stress profiles were categorized by PD severity groups (Table 1). The study population had an average age of 45 years (45.89 ± 11.29), with the majority being smokers (64.6%) and having a college-level education or higher (57.6%).

The study cohort, referred for dental examination, had a greater number of heavy drinking years and average drinks per day, lower household income, and a higher rate of being single and divorced/separated/widowed than the excluded cohort (See Supplementary Table S1). There were no notable differences in education status, ACE exposure, or perceived stress between these two groups.

All patients referred for dental examination were diagnosed with at least gingivitis/mild PD, while 73% had moderate to severe PD. Age was significantly associated with PD severity (*p* < .001), but other demographic variables did not show significant differences across PD severity groups. On average, patients reported moderate levels of perceived stress (22.78 ± 7.53) and had experienced one or more ACEs (42.84 ± 20.65).

### 3.2 DMFT estimate scores are higher in individuals with AUD who have severe PD

Total DMFT estimate scores were assessed across patients with gingivitis/mild, moderate, or severe PD. Total DMFT estimate scores were significantly associated with PD severity (*p* = .017; Supplementary Figure S1A), and patients with gingivitis/mild PD had significantly lower DMFT estimate scores compared to patients with severe PD (7.08 ± 8.03 *vs*. 12.93 ± 8.59, respectively; *p* = .012). No significant differences were observed in DMFT estimate scores between patients with moderate compared to severe PD (*p* = .277) nor between mild and moderate PD (*p* = .233; Supplementary Figure S1B). Furthermore, DMFT estimate scores did not differ across people who reported oral pain compared to those who did not (*p* = .296).

### 3.3 Increased PD severity was associated with a higher number of heavy drinking years

**Figure 3:**
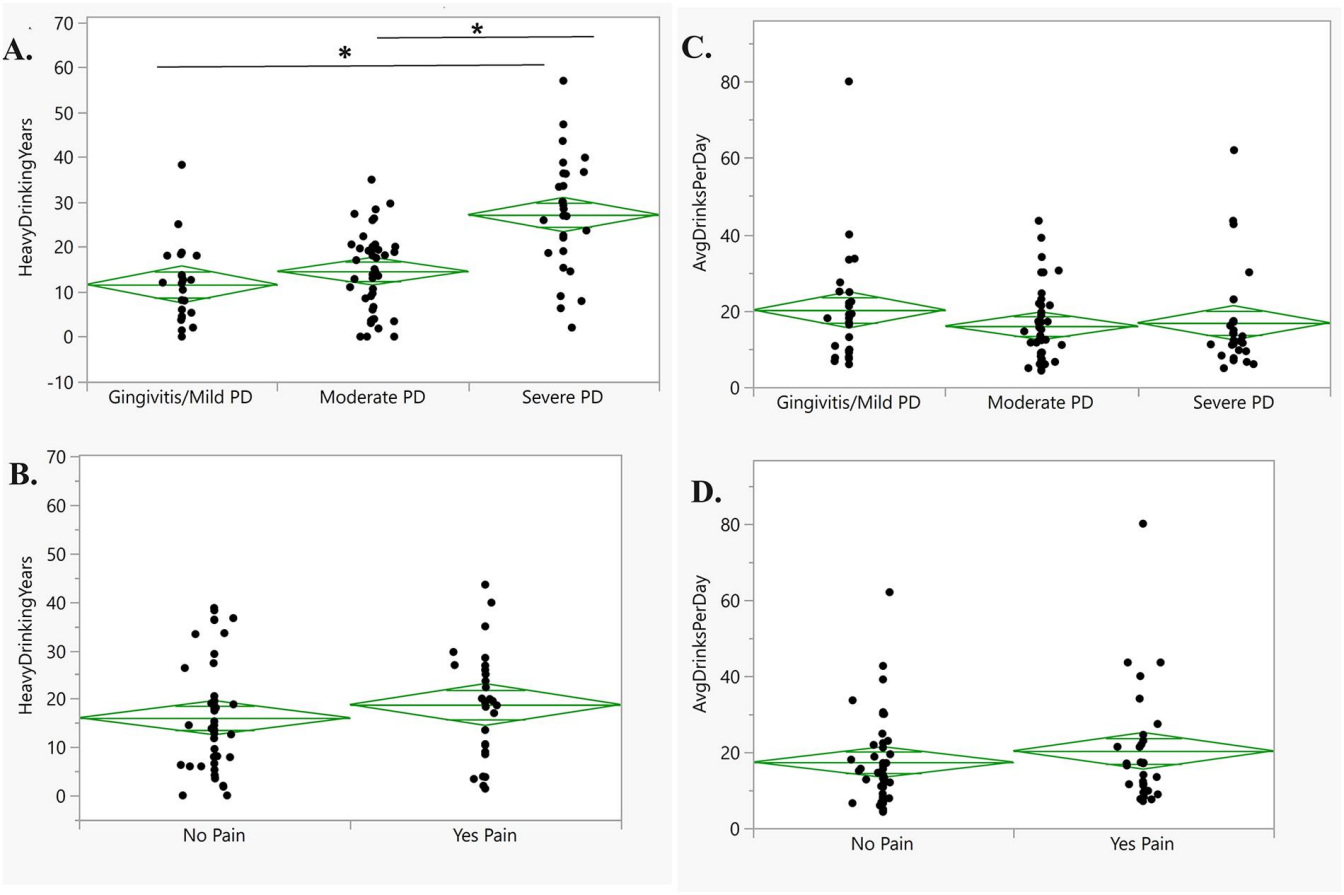
Alcohol Use variables association with oral health measures. Legend. Bivariate analyses comparing heavy drinking years and average drinks per day across PD severity and oral pain presence. **(A)** Heavy drinking years were significantly different across PD severity groups, i.e., heavy drinking years were significantly different between moderate and severe PD (*p* < .001) as well as between mild and severe PD (*p* < .001); **(B)** Heavy drinking years were not significantly different between subjects reporting the presence or absence of oral pain (*p* = .333); **(C)** Average drinks per day were not significantly associated with PD severity (*p* = .369) and **(D)** Average drinks per day were not significantly associated with oral pain presence (*p* = .351).

Alcohol use measures (i.e., heavy drinking years and average drinks per day in the 90 days before inpatient admission) were assessed in bivariate analyses across patients with mild, moderate, or severe PD. The number of years of heavy drinking was strongly associated with PD severity (*p* < .001; Figure 3A), but not with reported oral pain (*p* = .333; Figure 3B). For example, patients with more years of heavy drinking were more likely to be diagnosed with severe PD compared to those with moderate PD or gingivitis/mild PD [27.14 ± 12.87 vs. 11.61 ± 8.53 years [*p* < .001], and 27.14 ± 12.87 vs. 14.55 ± 8.86 years [*p* < .001], respectively]. However, the number of heavy drinking years did not significantly differ between patients with gingivitis/mild PD and those with moderate PD (*p* = .492). The average number of drinks per day in the 90 days before inpatient admission was not significantly associated with PD severity (*p* = .369; Figure 3C) nor with the presence of oral pain (*p* = .351; Figure 3D).

### 3.4 Perceived stress and ACEs are associated with oral health status in treatment-seeking individuals with AUD

#### 3.4.1 Perceived stress was higher in individuals with AUD who had gingivitis/mild PD

**Figure 4:**
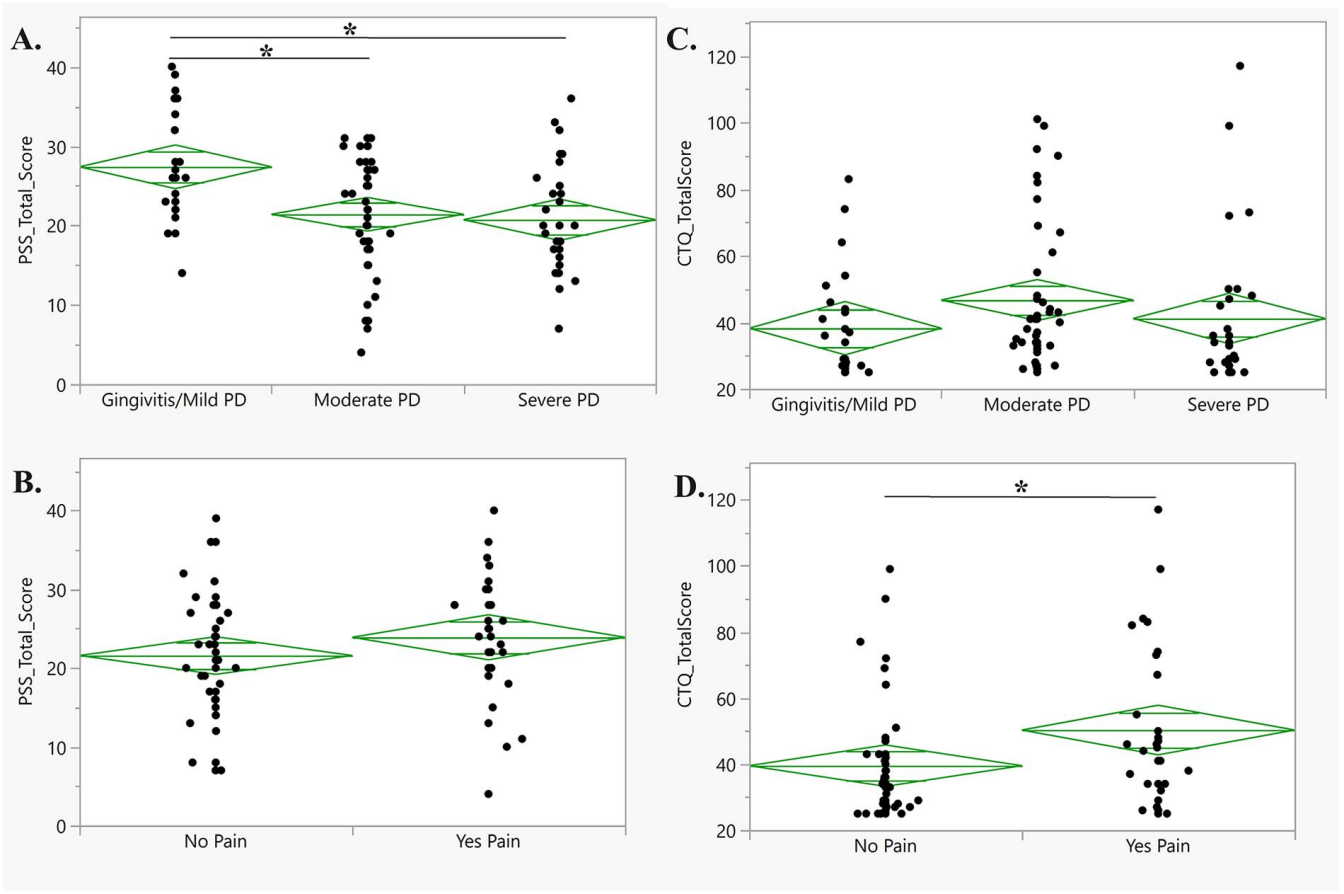
Current perceived stress and adverse childhood experience association with oral health measures. **(A)** Perceived stress total score for each patient (*y*-axis) stratified by PD. Horizontal lines indicate a significant *post-hoc* difference. Pairwise significance of current perceived stress was found between gingivitis/mild PD and severe PD (*p* = .002) as well as gingivitis/mild PD and moderate PD (*p* < .001). **(B)** Perceived stress total score for each patient (*y*-axis) stratified by oral pain presence. No significant difference was found between having oral pain and not having oral pain (*p* = .914). **(C)** ACEs for each patient (*y*-axis) were stratified by PD severity. No significant difference was found between mild, moderate, and severe PD status (*p* = .223). **(D)** ACEs for each patient (*y*-axis) were stratified by oral pain presence. Horizontal lines indicate a significant *post-hoc* difference. Significant differences in CTQ scores were found between having oral pain and not having oral pain (*p* = .031).

Perceived stress, measured by the PSS, was assessed across patients with mild, moderate, or severe PD. PSS significantly differed among PD severity groups (*p* < .001; Figure 4A). Patients with gingivitis/mild PD reported significantly higher PSS (27.38 ± 6.89) compared to patients with moderate PD (21.41 ± 7.24; *p* = .003) and severe PD (20.72 ± 6.97; *p* = .002). No significant differences were observed in PSS levels between patients with moderate compared to severe PD (*p* = .914; Figure 4A), or between patients with and without oral pain (*p* = .219; Figure 4B).

#### 3.4.2 ACEs were associated with oral pain presence

The number of ACEs, measured by the CTQ, was assessed across patients with gingivitis/mild, moderate, or severe PD. There was no significant difference in the number of ACEs among PD severity groups (*p* = .223; Figure 4C). However, patients who reported the presence of oral pain had significantly higher CTQ scores (50.30 ± 23.94) compared to patients who did not report oral pain (39.51 ± 17.96; *p* = .031; Figure 4D).

### 3.5 Alcohol intake characteristics are not associated with oral health when controlling for stress phenotype

#### 3.5.1 Perceived stress and ACE exposure have disparate relationships with PD severity

In the final model, age, household income, CTQ, and PSS scores were significant predictors of PD severity. After controlling for smoking status, age, heavy drinking years, household income, sex, and CTQ, for every one-unit increase in the PSS scale, the odds of a gingivitis/mild PD classification were approximately 1.19 times higher (95% C.I. = 1.046–1.359, *p* = .008) than the odds of having a moderate PD classification, and 1.18 times higher (95% C.I. = 1.022–1.374, *p* = .024) than the odds of having a severe PD classification in this study cohort (Table 2).

**Table 2:** Influence of stress and alcohol use on periodontal status.

PD severity group	Covariate	***β***	**SE**	***p*-value**	**Adjusted OR**	**95% CI**
Moderate	Intercept	−1.863	1.983	.347		
	Age	.085	.043	.**049**	1.088	(1.000, 1.184)
	Heavy Drinking Years	.048	.049	.327	1.049	(.953, 1.154)
	CTQ Total Score	.052	.025	.**036**	1.053	(1.003, 1.106)
	PSS Total Score	−.176	.067	.**008**	.839	(.736, .956)
	Household Income	.252	.138	.069	1.286	(.981, 1.687)
	Non-Smokers	−1.170	.847	.167	.310	(.059, 1.632)
	Female	.058	.782	.940	1.060	(.229, 4.906)
Severe	Intercept	−6.986	2.859	.015		
	Age	.206	.059	**<**.**001**	1.228	(1.095, 1.378)
	Heavy Drinking Years	.077	.054	.159	1.080	(.970, 1.201)
	CTQ Total Score	.044	.028	.113	1.045	(.990, 1.104)
	PSS Total Score	−.170	.075	.**024**	.844	(.728, .978)
	Household Income	−.018	.177	.921	.983	(.695, 1.389)
	Non-Smokers	−2.114	1.050	.**044**	.121	(.015, .946)
	Female	−1.772	1.098	.107	.170	(.020, 1.463)

PD severity reference category: gingivitis/mild PD; β, Beta coefficient; SE, standard error; OR, odds ratio; CI, confidence interval.

Values in bold indicate statistically significant *p* values.

#### 3.5.2 Perceived stress and ACE exposure are not associated with oral pain status after controlling for demographic variables

After controlling for smoking status, household income, sex, and age, neither PSS nor CTQ remained significantly associated with the presence of oral pain (OR = .979, 95% C.I. = .954–1.005, *p* = .118 and OR = .962, 95% C.I. = .897–1.032, *p* = .283, respectively; Table 3). However, after controlling PSS and CTQ, smokers had significant higher odds of experiencing oral pain (OR = 4.953, 95% C.I. = 1.386–17.702, *p* = .014) compared to non-smokers.

**Table 3:** Influence perceived stress and adverse childhood experiences on oral pain.

Covariate	**β**	**SE**	***p*-value**	**OR**	**95% CI**
Smoking	1.600	.649	.**014**	4.953	(1.386, 17.702)
CTQ Total Score	−.021	.013	.118	.979	(.954, 1.005)
PSS Total Score	−.038	.035	.283	.962	(.897, 1.032)
Household Income	−.083	.110	.455	.921	(.741, 1.144)
Female	−.193	.633	.760	.824	(.238, 2.851)
Age	−.031	.0,247	.212	.970	(.924, 1.018)

Oral pain reference category: No (reported oral pain). β, Beta coefficient; SE, Standard Error; OR, Odds Ratio; CI, Confidence Interval. No significant relationships were found between the oral health measure of DMFT estimate scores and alcohol use measures, perceived stress, or adverse childhood experiences (data not shown).

Values in bold indicate statistically significant *p* values.

## 4 Discussion

The purpose of this study was to examine relationships between ACEs, perceived stress severity, and measures of oral health in treatment-seeking individuals with AUD. The main findings of this retrospective analysis were that when controlling for clinically and significantly relevant covariates: 1) increased PSS scores were significantly associated with decreased PD severity, 2) increased ACE exposures were associated with an increased likelihood for moderate PD severity (compared to gingivitis/mild PD), but 3) duration of heavy drinking years and average drinks per day prior to inpatient admission was not associated with PD severity. These results suggest that stress evaluation may be an important factor to assess when creating a comprehensive risk evaluation for oral disease in individuals with AUD. Nevertheless, because this was a retrospective observational analysis, the findings should be interpreted as associations rather than evidence of causality, while also providing a basis for future prospective and mechanistic studies. The relationships among psychosocial stress, alcohol use, smoking, and oral health are likely bidirectional and may also be shaped by unmeasured behavioral and structural factors, including oral hygiene practices, diet, and access to dental care.

### 4.1 Seventy three percent of the individuals with AUD referred for dental examinations had moderate to severe PD

Between the years of 2017 through 2021, 258 patients were admitted to the NIH Clinical Center for inpatient treatment for AUD. Ninety-nine of these 258 patients were referred for a dental examination and had complete questionnaires assessing stress. In this study cohort of inpatients undergoing treatment for AUD that were referred for dental evaluation, many inpatients were male, a third had severe PD, and all had at least gingivitis/mild PD. Compared with patients who were not referred for dental examination, referred patients had a greater number of heavy drinking years and a higher average number of drinks per day; however, ACE exposure and perceived stress severity did not differ meaningfully between the two groups. Because oral health characteristics were only available in patients referred for dental examination, the study sample may have been enriched for individuals with more severe oral disease and therefore may not capture the full spectrum of alcohol- and stress-associated oral health outcomes in treatment-seeking individuals with AUD. These findings suggest that more individuals admitted for inpatient AUD treatment may benefit from referral for oral health and dental evaluation, and where feasible, broader referral of treatment-seeking individuals with AUD may help capture the full spectrum of oral and periodontal disease in this population.

### 4.2 Alcohol Use chronicity was not associated with increased PD severity or incidence of oral pain

After accounting for relevant covariates, alcohol use chronicity was not independently associated with PD severity, and alcohol use measures were not associated with oral pain in this sample. In the bivariate analyses, years of heavy drinking were significantly associated with PD severity, whereas average drinks per day prior to inpatient admission were not. In addition, neither heavy drinking years nor average drinks per day was associated with reported oral pain. However, when relevant covariates (i.e., age, smoking status, sex, household income, PSS, and CTQ scores) were included in the logistic regression model, the association between heavy drinking years and PD severity was no longer significant. These findings differ from previous research that chronic alcohol use strongly influences the progression of PD (18, 19, 55). Although confirmation in prospective research is needed, our results suggest that other variables may be equally or more important to consider when evaluating risk factors for PD in individuals with AUD. Access to oral health care services before inpatient treatment and oral health maintenance behaviors within this population may provide additional insight into factors that influence the relationship between alcohol use and oral disease. Continued research would also benefit from assessing environmental and structural factors, such as access to care and socioeconomic barriers, that may impact both AUD and oral health outcomes.

As expected, we found that severe PD was significantly associated with a higher DMFT estimate score when evaluating the relationship between objective oral health measures. This finding is consistent with the broader understanding that periodontal health and dentition are closely linked, such that worsening periodontal status often co-occurs with greater dentition-related disease burden (56, 57). In addition, these results are consistent with previous research in treatment-seeking individuals with AUD showing that moderate to severe PD was associated with higher DMFT estimate scores (55).

### 4.3 Periodontal disease severity had an inverse relationship with perceived stress

Results from this study did not support our hypothesis that higher perceived stress would be associated with increased PD severity or reported oral pain. In both the bivariate and adjusted analyses, perceived stress was inversely associated with PD severity, such that participants with gingivitis/mild PD reported higher PSS than those with moderate or severe PD. This finding is counter to prior literature, which has generally linked higher stress with worse periodontal outcomes (58) and poorer response to periodontal therapy (59). One possible explanation is that the PSS was administered at the time of admission to inpatient treatment and captures stress perceptions and reactions over the preceding 30 days. As such, the measure may have captured participants' immediate psychological response to treatment entry, alcohol withdrawal, or abrupt behavioral or environmental change rather than the longer-term subjective stress exposures more relevant to the development and progression of PD. This interpretation may be particularly relevant in individuals with AUD, in whom stress-related responses are heterogeneous and may be shaped by resilience, coping capacity, and broader affective functioning (60). In addition, recent psychometric research suggests that the PSS may partly reflect broader affective traits, such as neuroticism or negative emotionality, rather than stress perception alone (21, 60–62). Because individuals with AUD often report higher rates of anxiety- and neuroticism-associated symptoms (63, 64) these factors may have influenced PSS scores in ways not directly related to oral health status (21). Finally, the relatively small sample size and limited representation of early PD severity phenotypes may have also contributed to this unexpected association. Accordingly, this finding should be interpreted cautiously. Future prospective studies with repeated stress assessments and more granular characterization of PD will be important to clarify the relationship between perceived stress and oral health outcomes in individuals with AUD.

### 4.4 Oral pain was associated with smoking status but not alcohol intake or stress phenotype

Unlike PD severity, oral pain was not associated with perceived stress or ACE exposure in the adjusted analyses. Initially, there was a significant bivariate association between CTQ scores and oral pain, but this relationship lost significance when PSS scores, household income, sex, and smoking were included in the model. However, current smoking remained significantly associated with an increased likelihood of experiencing oral pain regardless of other covariates. Meta-analyses have shown that individuals with existing PD who smoke are at an increased risk for experiencing/reporting oral pain (65, 66), and a longitudinal study in older adults without AUD has shown similar results where smoking increasing the risk of oral pain to the extent that it interfered with activities of daily living (67). Additionally, individuals with AUD are twice as likely to smoke as those without AUD (68), highlighting smoking cessation as a crucial health behavior to target for improving oral health and oral-associated quality of life in this population.

Emphasizing smoking cessation alongside oral health-promoting behaviors, such as brushing and flossing, in inpatient treatment settings for individuals with AUD may support comprehensive oral health maintenance and help reduce the risk or progression of oral disease (69). Continuing research on the social factors that affect health and the external barriers to accessing interventions and treatments is essential for improving health outcomes in individuals with AUD and the general population. Specifically, gaining insight into how factors like insurance coverage, employment, and beliefs about oral health influence behaviors such as smoking and receiving preventive dental care will help develop strategies to improve oral health outcomes for diverse patient groups, including those with AUD.

### 4.5 Limitations and future directions

In this retrospective analysis of treatment-seeking individuals with AUD, we examined the relationships between perceived stress, ACE exposure, and oral health. However, several limitations should be considered when interpreting our findings. First, we were only able to include patients who were referred for dental evaluation, which likely biased the sample toward individuals with more apparent dental or periodontal issues or other oral-associated complaints. Additionally, the retrospective design resulted in missing data in some dental charts, further limiting the study sample as dental, periodontal, and oral pain assessments were required for the oral health variable characterization. The relatively small sample size may have reduced statistical power, limited subgroup comparisons, and decreased the generalizability of the findings. We were also unable to control for several potentially important confounders, including oral hygiene practices, diet, and access to dental care, because these data were not available in the clinical record. These factors are being evaluated in an ongoing study of a similar treatment-seeking cohort, which may help clarify their contribution to oral health outcomes in this population (70).

PD severity was classified retrospectively from clinician-documented diagnoses in routine dental records using dental progress notes and imaging for chart abstraction, rather than from a prospectively standardized research periodontal examination; because calibration and inter-rater reliability data were not available, misclassification cannot be excluded. Accordingly, these findings should be interpreted with caution. However, as an exploratory analysis intended to inform future hypothesis generation, this study provides important preliminary information about potential relationships between stress exposures and oral health in treatment-seeking individuals with AUD.

As most patients in the study cohort had moderate or severe PD, we merged participants with gingivitis and mild PD into a gingivitis/mild PD severity group to facilitate more balanced study groups due to low numbers of patients with a diagnosis of gingivitis (*n* = 8). Although this approach improved analytic stability, it may have obscured potentially meaningful distinctions between early periodontal phenotypes and reduced clinical specificity in our estimates. Accordingly, the findings for this grouped category should be interpreted conservatively. Future studies with larger samples should evaluate gingivitis and mild periodontitis separately. Similarly, the DMFT variable was based on retrospectively available clinical documentation and radiographic findings in a subset of the study cohort rather than a uniformly administered standardized research assessment; accordingly, we refer to this measure as a DMFT estimate, and findings involving this variable should be interpreted cautiously. Finally, the absence of biological and inflammatory biomarkers in this research limits our understanding of underlying mechanisms to which stress impacts oral health. Despite these limitations, our findings suggest that treatment seeking individuals with AUD could greatly benefit from dental referrals, enabling future research to capture differences in oral health and stress between patients with and without PD. Additionally, standardizing clinical documentation across patients will facilitate consistent information for clinical review and aid future epidemiological research on oral health and disease.

AUD remains a growing issue in the United States and globally, and beyond treatment-seeking individuals with AUD, approximately one-quarter of United States adults have untreated tooth decay (71). As tooth decay and PD become increasingly prevalent chronic conditions affecting both children and adults, the White House, the World Health Organization, and the National Institutes of Health have called for actions to address this escalating public health concern (71, 72). Data from this retrospective analysis suggest that increased referral of patients for dental examination and oral health management are integral to a comprehensive treatment plan in individuals with AUD. In addition to treatment-seeking individuals with AUD, coupling clinical dental measures (i.e., oral pain, DMFT estimate scores, and PD severity) with measures of stress as part of routine oral and dental examinations could provide valuable insight into psychosocial factors that predict PD severity across patient populations.

Quantifying and evaluating oral health maintenance behaviors was a missing factor in our retrospective analysis, as it was not captured in the original dataset. Although behavioral interventions have the potential to improve oral health outcomes, there is limited research on the frequency of oral health behaviors across populations or the obstacles to regularly practicing oral maintenance behaviors (73–75). Understanding patient demographics and environmental modifiers of oral health, along with oral care behaviors, could identify modifiable risk factors to reduce oral-associated disease in individuals with and without AUD. Developing and validating measures of oral health behaviors in individuals with AUD and similar high-risk populations would strengthen future research examining behavioral and psychosocial contributors to PD risk. More precise assessment of these behaviors could also support the identification of modifiable targets for intervention and help refine oral health education and prevention strategies in this population.

While we controlled for age, sex, smoking, and income, we were unable to account for other factors that may influence oral health, including diet, oral hygiene practices, and access to dental care, as these data were not available in this retrospective analysis. More broadly, many studies examining predictors or correlates of oral health across populations have not included detailed measurements of oral health maintenance behaviors. Nevertheless, the limited research that has assessed these behaviors suggests that oral health behavioral interventions may improve oral health outcomes (73, 74). Future research should prioritize the development and validation of measures to quantify oral health behaviors, particularly in individuals with AUD, and further examine inflammatory mechanisms linking oral and systemic health in this population (70, 76). Overall, this retrospective analysis provides preliminary information to inform our ongoing and future investigations, and highlights the potential relevance of stress exposures to oral health outcomes in treatment-seeking individuals with AUD.

## 5 Conclusion

In this retrospective sample of treatment-seeking inpatients with AUD referred for dental evaluation, moderate-to-severe PD was common. After adjustment for available covariates, heavy drinking years were no longer associated with PD severity, whereas psychosocial measures showed model-specific associations with periodontal outcomes. In the adjusted analysis, smoking, rather than perceived stress or childhood trauma, was associated with oral pain. These findings indicate that psychosocial factors may be relevant correlates of periodontal status in this population; however, the retrospective design, referred sample, chart-based abstraction of dental variables, and potential residual confounding preclude causal or predictive inferences. Accordingly, these results should be interpreted cautiously. Nevertheless, this study provides useful preliminary evidence to inform future prospective, hypothesis-driven research on psychosocial and behavioral correlates of oral health in individuals with AUD.

## Author's note

This research was supported by the Intramural Research Program of the National Institutes of Health (NIH). The contributions of the NIH authors are considered Works of the United States Government. The findings and conclusions presented in this paper are those of the authors and do not necessarily reflect the views of the NIH or the U.S. Department of Health and Human Services.

## Data Availability

The datasets presented in this study can be found in online repositories. The names of the repository/repositories and accession number(s) can be found below: Figshare Repository https://doi.org/10.6084/m9.figshare.26513245.v1.

## In Memoriam

Dedicated in memory of Delona Matthews.
