# Supplementary material for: Alcohol intake patterns, stress exposures and oral health in treatment seeking individuals with alcohol use disorder
Source: Front Oral Health. 2026 May 8;7:1736280. doi: 10.3389/froh.2026.1736280 (PMC13194103; doi:10.3389/froh.2026.1736280)
Supplement: Supplementary file 1 [file Datasheet1.docx]

Supplementary Material

# Supplementary Data

The dataset analyzed during the current study are available in the figshare data repository, and can be accessed at <https://doi.org/10.6084/m9.figshare.26513245.v1>

# Supplementary Figures and Tables

## Supplementary Tables

| **Supplemental Table S1. Demographic, Clinical, Stress and Alcohol-Associated Characteristics of Individuals Referred *vs*. Not Referred for Dental Examination** | | | |
| --- | --- | --- | --- |
| **Variables** | | **Referrals**  **(n = 99)** | **No Referrals**  **(n = 159)** |
| ***Sociodemographic Characteristics of Sample Population*** | | | |
| *Age (Mean ± SD)* | | 45.89 ± 11.3 | 45.10 ± 12.16 |
| *Sex, N (%)* | Male | 69 (69.7) | 100 (62.9) |
|  | Female | 30 (30.3) | 55 (34.6) |
|  | Not Reported | 0 | 4 (2.5) |
| *Marital Status, N (%)* | Single | 53 (53.5) | 94 (59.1) |
|  | Married | 10 (10.1) | 32 (20.1) |
|  | Divorced/Separated/ Widowed | 29 (29.2) | 19 (12) |
|  | Not Specified | 7 (7.1) | 14 (8.8) |
| *Education Level, N (%)* | Elementary School | 3 (3.0) | 2 (1.3) |
|  | Middle School |  | 3 (1.9) |
|  | High School | 39 (39.4) | 53 (33.3) |
|  | College | 49 (49.5) | 66 (41.5) |
|  | Graduate School or Higher | 8 (8.1) | 13 (8.2) |
|  | Not Reported |  | 22 (18.8) |
| *Household Income, N (%)* | < $5,000 | 27 (27.3) | 26 (16.4) |
|  | $5,000 - $19,000 | 25 (25.3) | 26 (16.4) |
|  | $20,000 - $49,999 | 25 (25.3) | 39 (24.5) |
|  | $50,000 - > $100,000 | 22 (22.2) | 46 (28.9) |
|  | Not Reported |  | 22 (18.8) |
| *Smoking Status, N (%)* | Smoker | 64 (64.6) | 93 (58.5) |
|  | Non-smoker | 35 (35.4) | 44 (27.7) |
|  | Not Reported |  | 22 (18.8) |
| ***Stress Characteristics of Sample Population*** | | | |
| *PSS Total Score (Mean ± SD)* | | 22.78 ± 7.53 | 22.52 ± 7.26 |
| *CTQ Total Score (Mean ± SD)* | | 42.84 ± 20.65 | 44.66 ± 17.80 |
| ***Alcohol Use Profile of Sample Population*** | | | |
| *Average Drinks Per Day (Mean ± SD)* | | 17.34 ± 12.15 | 14.63 ± 10.05 |
| *Heavy Drinking Years (Mean ± SD)* | | 17.52 ± 11.87 | 14.33 ± 9.98 |

Abbreviations: CTQ= Child Trauma Questionnaire, PD= Periodontal Disease, PSS= Perceived Stress Scale, SD= Standard Deviation.

| **Supplemental Table S2.** Mild Periodontal Disease Sub Diagnoses Distribution | | |
| --- | --- | --- |
|  | **Sub Diagnosis** | **N (%)** |
| Mild Periodontal Disease | Mild Gingivitis | 5 (19.2) |
|  | Moderate Gingivitis | 3 (11.5) |
|  | Mild Periodontitis | 18 (69.2) |

## Supplementary Figures

**Supplementary Figure S1. Associations of Decayed, Missing, and Filled Teeth (DMFT) Estimate Scores and Measures of Oral Health**


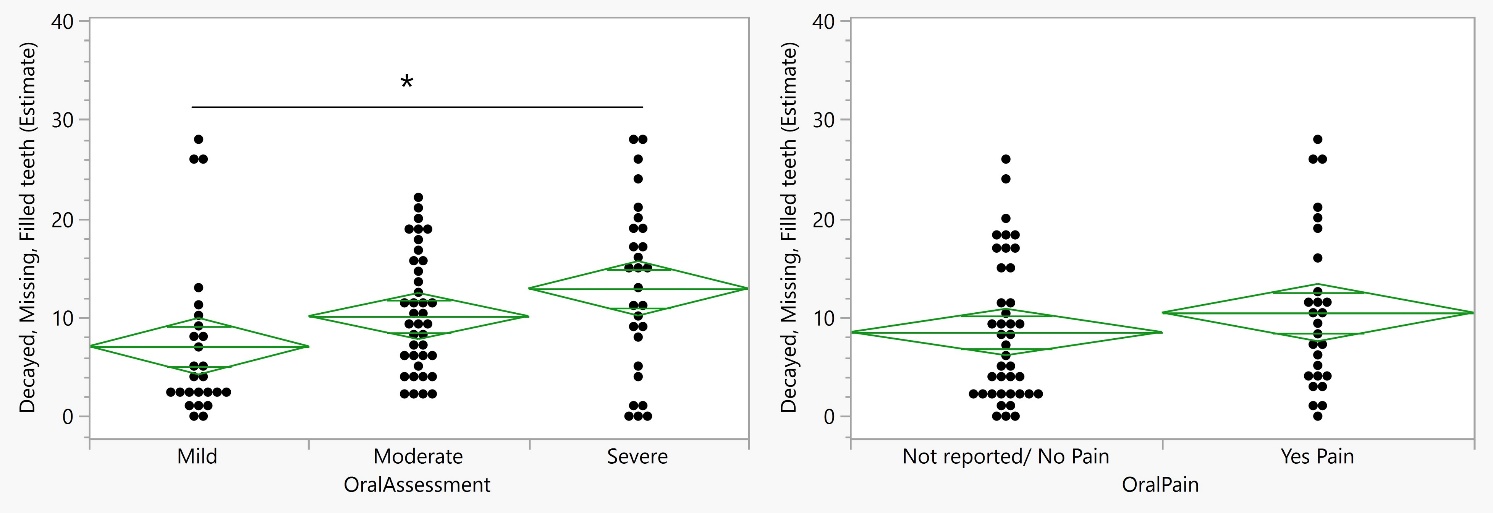


**Supplementary Figure S1.** A) Total DMFT estimate for each patient (y-axis) was stratified by periodontal disease (PD) severity. Significant differences in DMFT estimate scores were found between PD severity groups (*p* = .017). Oral pain presence for each patient (y-axis) was stratified by PD severity. B) Total DMFT estimate scores were not significantly different between individuals who reported oral pain and individuals who did not report oral pain (*p* = .296). *post-hoc group differences *p*<.05.
